# A comprehensive national audit of radiotherapy retreatment numbers, sites and indications

**DOI:** 10.2340/1651-226X.2025.43825

**Published:** 2025-07-09

**Authors:** Morten Nielsen, Mai-Britt Linaa, Vibeke Nordmark Hansen, Laura Patricia Kaplan, Mikkel Drøgemüller Lund, Martin Skovmos Nielsen, Wiviann Ottosson, Cécile Peucelle, Laura Ann Rechner, Heidi S. Rønde, Tine Schytte, Weronika Maria Szejniuk, Rebecca Jean Tobin, Lone Hoffmann, Ane Appelt

**Affiliations:** a Department of Oncology, Odense University Hospital, Odense, Denmark; b Department of Oncology, Aarhus University Hospital, Aarhus, Denmark; c Department of Oncology, Centre for Cancer and Organ Diseases, Copenhagen University Hospital – Rigshospitalet, Copenhagen, Denmark; d Royal Marsden NHS Foundation Trust, Sutton, Surrey, UK; e Department of Oncology and Palliative Units, Zealand University Hospital, Næstved, Denmark; f Department of Oncology, Vejle Hospital, University Hospital of Southern Denmark, Vejle, Denmark; g Department of Oncology & Clinical Cancer Research Center, Aalborg University Hospital, Aalborg, Denmark; h Department of Clinical Medicine, Aalborg University, Aalborg, Denmark; i Department of Oncology, Radiotherapy Research Unit, Copenhagen University Hospital – Herlev and Gentofte, Herlev, Denmark; j Danish Centre for Particle Therapy, Aarhus University Hospital, Aarhus, Denmark; k Department of Clinical Research, University of Southern Denmark, Odense, Denmark; l Department of Clinical Medicine, Aarhus University, Aarhus, Denmark; m Leeds Institute of Medical Research, University of Leeds, Leeds UK

**Keywords:** Reirradiation, repeat irradiation, retreatment, radiotherapy, national audit

## Abstract

**Background and purpose:**

Reirradiation has seen increased interest and clinical use; however, robust data on patient numbers and treatment indications are missing. As a precursor to a prospective national reirradiation registry, a comprehensive national audit of reirradiation was performed.

**Patients/materials and methods:**

Radiotherapy retreatment courses in 2023 were audited by all (eight) radiotherapy centres in Denmark. Six centres extended the evaluation to include 2021–22, and three of these also evaluated preceding years. Reirradiation was defined according to the ESTRO/EORTC consensus (i.e. treatment volume overlap or cumulative dose toxicity risk) using 3 months threshold between the primary and reirradiation courses. Reirradiation courses were further stratified into curative/ablative and palliative treatments by prescription dose.

**Results:**

The total number of radiotherapy patients at Danish centres in 2023 was 17,424. Of these, 3,163 received retreatment, including 1,471 reirradiation courses (1,035 palliative; 436 curative/ablative). From 2014 to 2023, absolute numbers for both retreatment and reirradiation increased. We found large variation in prescription doses and fractionation schedules used for reirradiation. Widely used palliative prescriptions were 8Gy/1 fraction (F), 20Gy/4F and 30Gy/10F; stereotactic prescriptions of 20Gy/1F or 27Gy/3F in brain and 45Gy/3F in lung; and a variety of curative treatments schedules. Palliative reirradiations were primarily thoracic (29%), spine (25%), and abdominal/pelvic (22%) and curative/ablative reirradiations were primarily breast (29%) and lung stereotactic (23%).

**Interpretation:**

This is the first comprehensive national audit of reirradiation, demonstrating an increasing number of patients being treated, using a wide variety of dose prescriptions and fractionation schedules.

## Introduction

The number of long-term cancer survivors at risk of relapse or secondary tumours is rising. This, along with advances in radiotherapy treatment planning and delivery, leads to an increase in patients returning for a second (or even third, fourth, or more) course of radiotherapy [1–7]. However, broadly accepted guidelines for clinical reirradiation management and decision making are at present lacking. This contributes to highly heterogeneous practices regarding patient selection, dose prescription, and treatment planning and delivery [8, 9].

Published studies on reirradiation are largely heterogeneous regarding both patient populations and dose prescriptions, and often comprise rather small numbers of patients. Results from single-centre studies may be less generalisable due to inter-institutional differences in both practices and patient cohorts. The generation of proper evidence to support broad guidelines requires larger-scale prospective studies. Such studies, in turn, are infeasible without first homogenising reirradiation practices among trial participants.

Current utilisation of reirradiation is not well documented, and data on patient selection, numbers and characteristics, as well as treatment indications, are scarce. Willmann et al. reported an international pattern of care survey in 2023, which provided an indication of practice amongst 371 physicians from 55 countries [10]. This was not a systematic practice audit, however, but a self-selected subset of clinicians answering an open survey. No previous studies have provided a complete account of nation-wide reirradiation patient numbers.

In preparation for a national Danish reirradiation registry study, the aim of this work was to perform a comprehensive audit of current reirradiation practices as well as their development in recent years.

## Patients/materials and methods

Radiotherapy retreatment courses in 2023 were audited by all (eight) radiotherapy centres in Denmark, including one proton facility. Six centres extended the evaluation to include 2021–22. As further extensions, one centre included 2014, one centre included 2015, 2017, and 2019, and one centre included 2014, 2015, 2017 and 2019. This selection of extensions was based on available resources and the time necessary for data extraction, which varied between centres.

The number of unique patients receiving radiotherapy, as well as the number of retreated patients, was counted for each year in each centre. This approach meant that each unique patient was only counted once each year, but could be counted again in another year if further retreatment was delivered. For the purpose of this study, an interval of 3 months between the first course and subsequent courses of radiotherapy was required in order for these to count as retreatment.

For retreated patients, each radiotherapy course was evaluated according to the ESTRO-EORTC Reirradiation Consensus Statement, published in 2023, which established a common ontology for repeated radiotherapy treatments [1]. It defines four subgroups of retreatment to facilitate communication and comparisons:

- Reirradiation
  - Type 1: New course of radiotherapy that has geometrical overlap with the irradiated volume of previous courses.
  - Type 2: New course of radiotherapy, with concerns of toxicity from the cumulative doses without overlap of irradiated volumes.
- Repeat organ irradiation: New course of radiotherapy to a previously irradiated organ but without overlap of the irradiated volumes and without concerns for toxicity from cumulative doses.
- Repeat irradiation: New course of radiotherapy to an organ that has not been irradiated, without overlap of irradiated volumes, and without concerns for toxicity from cumulative doses.

In this study, patients and treatment courses were further grouped as either ‘reirradiation’ (type 1 or 2) or ‘other retreatment’ (repeat organ irradiation or repeat irradiation). Treatment courses were included in the ‘reirradiation’ cohort if they fulfilled the ESTRO-EORTC reirradiation definition above. In practice, this was interpreted as any treatment where there was direct overlap of treated volumes or where the previous plan was considered in planning or decision making for the retreatment. As a specific case, patients receiving contralateral tangential breast retreatment were universally classified as reirradiation, because targets and beam angles are always considered and usually adjusted to account for previous fields. Note that a patient retreated in a particular year could have several reirradiation treatment courses and that each treatment course was counted separately.

One centre had identified and annotated reirradiations prospectively in their record and verify system to facilitate subsequent retrieval, while the other seven centres retrospectively extracted data from record and verify and/or treatment planning systems. The exact methodology applied to identify reirradiation varied between centres depending on the record and verify system, treatment planning system, naming convention, etc. (Table 1).

**Table 1:** Methodology used by the participating centres to audit reirradiations at least 3 months after the de novo radiotherapy.

Centre 1	A gross list of patients was extracted from the record and verify system where more than 3 months had passed from the date of diagnosis to the start of a treatment course. Treatments before 2018 were manually audited to determine whether the retreatments were reirradiation. From 2018 onward, a treatment plan naming convention identified whether a treatment course was a reirradiation.
Centre 2 (Proton facility)	Patients in formal reirradiation study protocols were directly identified from protocol databases. Other retreatments were manually audited to determine whether the present treatment planning was a reirradiation.
Centre 3	A gross list of patients was extracted from the record and verify system with more than one treatment course at least 3 months apart. Local naming conventions allowed identification of treatments localised in proximity to previous treatment sites. Manual audit of these treatment plans allowed identification of type 1 and type 2 reirradiations.
Centre 4	A gross list of patients was extracted from the record and verify system with more than one treatment course at least 3 months apart or where relevant keywords were present in the clinical notes. Manual audit of the list allowed the identification of type 1 and type 2 irradiations, repeat organ irradiation or repeat irradiation.
Centre 5	A gross list of patients was extracted from the record and verify system, consisting of patients where the start of two treatment courses was separated by more than 12 weeks or the patient was prospectively flagged as a retreatment. This list was expanded with patients for whom isodoses from a previous treatment were imported into the treatment planning system. Manual audit of the list allowed the identification of type 1 and type 2 irradiations, repeat organ treatment or repeat irradiation, with 3 months separation between the first course of and any subsequent course of radiotherapy.
Centre 6	A gross list of patients with more than one treatment course at least 3 months apart in the record and verify system was extracted. A manual audit of these patients in the treatment planning system allowed identification of type 1 and type 2 irradiations.
Centre 7	Treatment courses in the record and verify system were numbered consecutively at the time of treatment planning. A gross list of patient treatments with course numbers 2 or higher was created. Manual audit allowed identification of type 1 and type 2 irradiations, repeat organ treatment or repeat irradiation.
Centre 8	Extraction from the record and verify system identified repeat radiotherapy treatments. Further extraction from treatment planning system identified treatment plans where isodose curves from previous treatments were imported. As per institutional practice, this was only done for cases where it was deemed necessary to consider previous irradiation; hence a reirradiation.

Reirradiation treatment courses were categorised by anatomical site and by prescribed dose and fractionation. The treatments were stratified into curative/ablative treatments and palliative treatments by prescribed dose, with prescribed doses of less than 40 Gy EQD2 (α/β = 10) considered as palliative, except for the following specific cases:

- Reirradiation of lymphomas and sarcomas, stereotactic reirradiation, reirradiation of benign cases, whole body reirradiation and preoperative irradiation of rectal cancer were always considered curative/ablative regardless of the prescribed dose.
- Reirradiation with 39 Gy/13 F was used as palliative reirradiation, primarily in the thoracic region.

## Results

The total number of patients treated with radiotherapy at all Danish centres in 2023 was 17,424. Of these, 3,164 received at least one retreatment course, of which 1,471 represented reirradiations of type 1 or type 2; 436 with curative/ablative intent and 1,035 with palliative intent (Table 2).

**Table 2:** Distribution of treatment sites for the year 2023 for all centres.

Reirradiations in 2023	Centre 1	Centre 2	Centre 3	Centre 4	Centre 5	Centre 6	Centre 7	Centre 8	Total
Number of patients	3,521	316	1,607	1,771	2,338	3,069	1,626	3,176	17,424
Number of retreatment patients^†^	735	38	231	174	350	662	323	651	3,164
Palliative reirradiation RT Courses Total	306	1	97	73	92	261	87	118	1,035
Spine	57	0	18	14	20	89	12	51	261 (25%)
Brain	10	0	7	1	13	13	7	9	60 (6%)
Head and Neck	32	0	15	2	13	15	8	5	90 (9%)
Thoracic	112	0	23	28	17	76	27	15	298 (29%)
Abdomen/pelvic	84	0	21	18	28	56	27	2	236 (23%)
Extremities	10	0	10	4	1	10	6	1	42 (4%)
Breast	0	0	0	6	0	0	0	0	6 (1%)
Other*	1	1	3	0	0	2	0	35	45 (4%)
Curative/ablative reirradiation RT Courses Total	72	37	24	48	103	76	36	40	436
Brain	1	1	0	0	0	0	0	2	4 (1%)
Head and Neck	7	0	1	0	7	2	6	3	26 (6%)
Lung	4	0	0	0	4	2	6	1	17 (4%)
Oesophagus	4	0	0	0	2	0	0	1	7 (2%)
Breast	24	16	2	25	15	13	23	13	131 (30%)
Pelvic	4	18	1	0	11	1	1	5	41 (9%)
Sarcoma	2	0	0	0	0	2	0	0	4 (1%)
Lymphoma	1	0	0	0	2	0	0	1	4 (1%)
Benign	0	0	3	0	0	0	0	0	3 (1%)
Whole body irradiation	0	0	0	0	0	0	0	1	1 (<1%)
Other non-stereotactic RT*	0	2	0	0	0	0	0	3	5 (1%)
Brain Stereotactic RT	7	0	7	0	19	42	0	2	77 (18%)
Lung Stereotactic RT	14	0	9	23	39	13	0	4	102 (23%)
Bone Stereotactic RT	4	0	1	0	3	0	0	0	8 (2%)
Abdominal Stereotactic RT	0	0	0	0	1	1	0	3	5 (1%)
Other Stereotactic RT*	0	0	0	0	0	0	0	1	1 (<1%)

RT: radiotherapy.

Centre 2 is a proton facility.

† Total of all patients receiving reirradiation, repeat organ irradiation, and repeat irradiation.

* ‘Other’ means ‘Data missing on organ site’. Note that any given patient may have received more than one reirradiation course, and thus the total number of reirradiations can exceed the number of retreatment patients.

Disregarding centre 2 (which is a proton therapy facility and thus treat very select patients), the number of patients receiving radiotherapy treatment in 2023 varies between centres from 1,607 at centre 3 to 3,521 at centre 1 (Table 1); a variation of approximately a factor two from the smallest to the largest radiotherapy centre. The fraction of these patients receiving at least one course of radiotherapy retreatment also varies across centres, from 10% for centre 4 to 21% for centres 1 and 6. The number of reirradiation courses relative to the total number of patients varies from 5% in centre 8 to 12% in centres 2 and 6.

**Figure 1:**
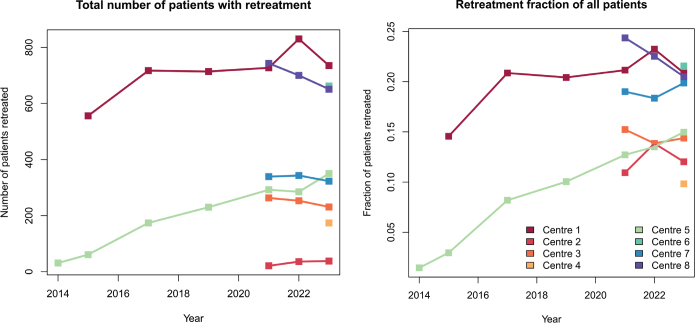
The total number of patients undergoing retreatment (reirradiation, repeat organ irradiation and repeat irradiation) for each year and for each radiotherapy centre; absolute numbers (upper pane) and as a fraction of all patients receiving radiotherapy at the centre (lower pane).

**Figure 2:**
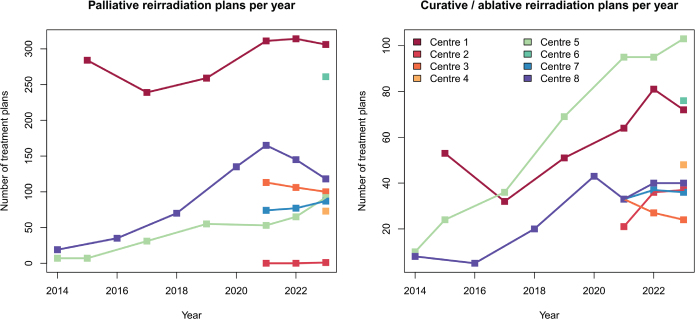
The total number of reirradiation courses (type 1 and type 2 reirradiation) for each year and for each radiotherapy centre; palliation courses (upper pane) and curative/ablative courses (lower pane).

From 2014 to 2023, the number of retreated patients and the fraction of all treated patients they constitute (Figure 1) as well as the number of palliative and curative reirradiation treatment plans generally increased (Figure 2).

**Figure 3:**
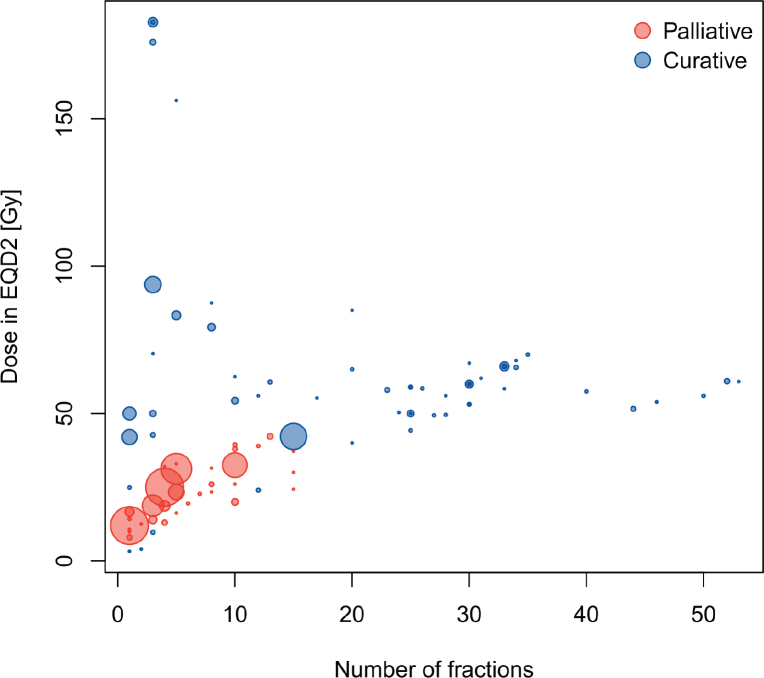
Distribution of prescription doses and fractions. The area of each marker is proportional to the number of courses utilising the prescription/fractionation.

The audit found large variations in the dose prescriptions and fractionation schedules used for reirradiation. In 2023, 33 different dose prescriptions/fractionations were used for palliations, and 58 different dose prescriptions/fractionations were used for curative/ablative reirradiations; with a large variation in the frequency of use (Figure 3). Across all years, 61 different prescriptions were used for palliations, while 104 different prescriptions were used for curative/ablative treatments. Certain prescriptions were widely used by all centres over the years, such as 8 Gy/1 fraction (F), 20 Gy/4 F, 30 Gy/10 F for palliative reirradiations; 20 Gy/1 F or 27 Gy/3 F in brain stereotactic reirradiations; 45 Gy/3 F in stereotactic oligometastatic lung reirradiations; and a variety of curative treatments to full definitive doses adhering to national guidelines or trials.

Palliative reirradiations were primarily thoracic (29%), spine (25%), and abdominal/pelvic (22%); while curative/ablative reirradiations were primarily breast (30%), lung stereotactic treatments (23%) and brain stereotactic treatments (18%) (Table 2).

## Discussion and conclusion

This study provides a comprehensive national audit of radiotherapy retreatment frequencies, sites, and prescriptions. It was found that an increasing number of patients are being retreated with radiotherapy and an increasing number of reirradiation treatment are delivered in Denmark over the years, and that dose prescription and fractionation schemes vary widely in reirradiation. While this result might seem intuitive, published data to evidence this have been scarce.

Willmann et al. [10] conducted an international cross-sectional review investigating the patterns of care of reirradiation for different anatomical sites. This highlighted the varying international practices with little consensus. Nieder et al. [1, 4] reviewed published randomised trials involving reirradiation, while Willmann et al. reviewed ongoing prospective trials involving reirradiation. In both cases, it was emphasised that large trials on reirradiation are challenging due to small patient populations, variability in practices, and a lack of generally accepted guidelines. Paradis et al. [6] has very recently reported an extensive and comprehensive single-institution experience on developing a framework for clinical decision making on reirradiation. All of these publications highlighted the need to combine data across institutions and regions and included a call for prospective and consistent data collection for these patients. To facilitate this, the ESTRO-EORTC consensus guideline [8] proposed terms and definitions for an ontology in the field of retreatment by radiotherapy.

The challenges in the procurement of data, as presented in the present audit, are highlighted by the fact that the details of institutional database reviews vary considerably (Table 1). Even with our current comprehensive data collection approach, it should be assumed that data are incomplete: Differences in data procurement procedure between centres likely lead to differences in data completeness. Additionally, patients who have received radiotherapy at two different facilities may not have been accurately flagged by the record and verify system search strategies used by many of the participating centres. Although this likely represents a small number of patients (due to the highly centralised nature of the Danish health care system), this should not be underestimated.

Nevertheless, our results represent the first comprehensive and robust estimate of reirradiation patient numbers. This was facilitated by:

- A national single-payer health care system with centralised patient identification and related databases
- The recent ESTRO-EORTC consensus article on reirradiation and repeat irradiation definitions, to provide a common ontology
- A formalised national collaboration through the Danish Comprehensive Cancer Center, to ensure participation by all centres.

To significantly increase future completeness of data, that is, identify all reirradiated patients, these should be prospectively collected by all centres; ideally by a strictly formalised procedure, for example, as described by Paradis et al. [6].

The fraction of patients receiving at least one course of radiotherapy retreatment ranges from 10% for centre 4 to 21% for centre 1. The number of reirradiation courses relative to the total number of patients varies from 5% in centre 8 to 12 % in centres 2 and 6. This is in line with the rate reported by Paradis et al. [6], especially when it is considered that the systematic framework of that study probably led to a much more complete record of reirradiated patients than what can be obtained in the retrospective audit used by most centres in this study. It is observed from the numbers in Table 2 that the absolute size of the auditing centres does not correlate with the relative number of retreatments or reirradiations.

As demonstrated by the data in Table 2, it is not just patient numbers and reirradiation rates that vary between centres. The composition of reirradiated tumour sites also varies considerably. For example, centres 1 and 6 referred a large number of patients for spine and thoracic palliations, whereas centres 4 and 5 delivered a large number of lung stereotactic reirradiations. Centres 5 and 6 had relatively large numbers of brain palliations and brain stereotactic reirradiations, whereas centre 1 had relatively few cases. The variations described may be due to differences in institutional practices, both in the choices between radiotherapy, systemic treatment, and surgery, and in the choices of dose prescription when radiotherapy is used in a retreatment setting. It should be noted that centre 7 does not perform any stereotactic treatments at all but refers these patients to other centres. These comparisons, and any conclusions drawn from them, are limited by the differences in data procurement procedures across centres.

Many different prescriptions were used for reirradiations in the auditing centres in 2023 (Figure 3). However, we did not audit variation in prescriptions and fractionations for de novo treatments (i.e. treatments which are not reirradiations, repeat organ irradiations or repeat irradiations) in the same period. Thus, it is not possible to conclude to what degree this variation is related to a reirradiation scenario, or whether this reflects a natural variation also for de novo treatments. However, national guidelines designed by the Danish Multidisciplinary Cancer Groups standardise the dose prescriptions for de novo treatments to some extent; while this is not (yet) the case for most reirradiation scenarios.

The division of reirradiations into curative/ablative and palliative treatment was based on planned dose, with consideration of the diagnosis and treatment target, and informed by current clinical practice in Denmark. However, it is difficult in a retrospective study to make a definite determination of what the treatment intent was at the time of treatment, and, in any case, it was beyond the scope of this study to do a full patient chart examination to obtain this information. Furthermore, some reirradiation cases involve suspected oligo-metastatic patients, and the division into curative and palliative intent may be blurred for such cases. Here, we decided to group all ablative treatments with other curatively intended reirradiation, even when the aim of the treatment might have been local control but not aiming for a fully curative treatment.

The present audit was conducted in support of an upcoming prospective national reirradiation registry ‘Prospective Registry for Oncologic Reirradiation’ (PRIOR), which will aim to include data on all retreatments aiming for local control or extended overall survival. Our findings will inform registry protocol and database design and future data from the registry may help investigate some of the inter-centre differences observed in the present audit.

In conclusion, this study presents a comprehensive national audit of radiotherapy retreatment frequencies, sites, and prescriptions. It was found that an increasing number of patients are receiving reirradiation in Denmark, and that dose prescription and fractionation schemes vary widely in reirradiation. The upcoming prospective national registry PRIOR follows the expressed need for further systematically collected data on reirradiation treatments.

## Data Availability

The clinical data underlying the audit numbers cannot be shared.

## References

[1] Nieder C, Langendijk JA, Guckenberger M, Grosu AL. Prospective randomized clinical studies involving reirradiation: lessons learned. Strahlenther Onkol. 2016 Oct;192(10):679–86. 10.1007/s00066-016-1024-6 27534408

[2] Bryant AK, Banegas MP, Martinez ME, Mell LK, Murphy JD. Trends in radiation therapy among cancer survivors in the United States, 2000–2030. Cancer Epidemiol Biomarkers Prev. 2017 Jun;26(6):963–70. 10.1158/1055-9965.EPI-16-1023 28096199

[3] Christ SM, Ahmadsei M, Wilke L, Kuhnis A, Pavic M, Tanadini-Lang S, et al. Long-term cancer survivors treated with multiple courses of repeat radiation therapy. Radiat Oncol. 2021 Oct 30;16(1):208. 10.1186/s13014-021-01934-y 34717664 PMC8557578

[4] Nieder C, Willmann J, Andratschke NH. Prospective randomized clinical studies involving reirradiation: update of a systematic review. Strahlenther Onkol. 2023 Sep;199(9):787–97. 10.1007/s00066-023-02118-1 37500926 PMC10449695

[5] Ahmadsei M, Christ SM, Kroese TE, Kuhnis A, Willmann J, Balermpas P, et al. Efficacy and safety analysis in metastatic cancer patients treated with multiple courses of repeat radiation therapy. Clin Transl Radiat Oncol. 2023 Nov;43:100687. 10.1016/j.ctro.2023.100687 37867613 PMC10589769

[6] Paradis KC, Mayo C, Matrosic CK, Prisciandaro JI, Rosen BS, Allen SG, et al. Reirradiation special medical physics consultations: lessons learned from nearly 3000 courses of treatment. Int J Radiat Oncol Biol Phys. 2025 Mar 9 [Online ahead of print]. 10.1016/j.ijrobp.2025.03.002 40068779

[7] Mayo CS, Appelt AL, Paradis KC, Dawson LA, Andratschke N, Vasquez Osorio EM, et al. Joining forces to advance reirradiation: establishing the reirradiation collaborative group. Int J Radiat Oncol Biol Phys. 2025;122(2):234–40. 10.1016/j.ijrobp.2025.01.038 40088225

[8] Andratschke N, Willmann J, Appelt AL, Alyamani N, Balermpas P, Baumert BG, et al. European Society for Radiotherapy and Oncology and European Organisation for Research and Treatment of Cancer consensus on re-irradiation: definition, reporting, and clinical decision making. Lancet Oncol. 2022 Oct;23(10):e469–78. 10.1016/S1470-2045(22)00447-8 36174633

[9] Armstrong S, Hoskin P. Complex clinical decision-making process of re-irradiation. Clin Oncol (R Coll Radiol). 2020 Nov;32(11):688–703. 10.1016/j.clon.2020.07.023 32893056

[10] Willmann J, Appelt AL, Balermpas P, Baumert BG, de Ruysscher D, Hoyer M, et al. Re-irradiation in clinical practice: results of an international patterns of care survey within the framework of the ESTRO-EORTC E(2)-RADIatE platform. Radiother Oncol. 2023 Dec;189:109947. 10.1016/j.radonc.2023.109947 37806559

